# Considerations on the Life Cycle of *Laminosioptes cysticola* (Vizioli, 1870) Based on a Natural Infestation in Two Laying Hens

**DOI:** 10.3390/ani15142024

**Published:** 2025-07-09

**Authors:** Iolanda Moretta, Simona Principato, Giuseppe Giglia, Elvio Lepri, Mario Antonello Principato

**Affiliations:** 1 Department of Veterinary Medicine, University of Perugia, Via San Costanzo, 4, 06126 Perugia, Italy; giglia.giuseppe.93@gmail.com (G.G.); elvio.lepri@unipg.it (E.L.); mario.principato@virgilio.it (M.A.P.); 2 Urania Research Centre, Via del Mattatoio Vecchio 7, 06063 Magione, Italy; urania@edpa.it

**Keywords:** poultry mites, fowl cyst mite, nodular acariasis

## Abstract

*Laminosioptes cysticola* (Vizioli, 1870), a tissue-dwelling mite responsible for nodular acariasis in birds, was identified from two hens reared in a rural backyard flock in Umbria, Italy. Adult mites were found in the subcutaneous tissue and on the serosal surface of various internal organs. Larval and first- and second-stage nymphal forms were observed beneath the skin and near the trachea and esophageal serosa. By comparing the existing literature with that reported in the present study, we propose a hypothetical reconstruction of the parasite’s life cycle. It is postulated that the entry of *L. cysticola* occurs through the cervical skin, where adults mate and larviparous females give birth to larvae. These larvae migrate into the loose connective tissues surrounding the trachea and esophagus, where they develop into nymphs. The immature forms then progress along the esophagus and trachea to reach the thoracic and abdominal cavities, colonizing the serosal surfaces of visceral organs. It remains unclear whether, or how, the mites return to the subcutaneous tissues to complete their maturation. Senescent specimens degenerate within the subcutis, where they are encased by a granulomatous inflammatory reaction that leads to the formation of characteristic calcified nodules.

## Simple Summary

In two adult hens from a rural farm, an infestation by *Laminosioptes cysticola* was diagnosed by necropsy and parasitological examination. It is a mite that colonizes the subcutaneous tissue and various visceral serosal surfaces, causing the formation of small nodules that tend to calcify over time. This is a relatively unknown parasite, whose biological cycle is still not exactly known. For this reason, by combining our observations—derived from the meticulous parasitological examination of the parasites present in various anatomical regions—with the information present in the literature, we have hypothesized a possible life cycle, highlighting the entry point in the subcutaneous tissue, the migrations carried out by the immature and adult forms, and the possible methods of diffusion within the farm.

## 1. Introduction

*Laminosioptes cysticola* (Vizioli, 1870) is a mite belonging to the family Laminosioptidae, known to colonize the subcutaneous tissue of a wide range of bird species, particularly Galliformes (chickens, pheasants, and turkeys) and Columbiformes (pigeons and doves). Infestation with this parasite results in a chronic nodular dermatosis known as “nodular acariasis”. Although reported across various geographic regions, *L. cysticola* remains rarely documented. In many of the available reports, diagnostic confirmation has been partial or inadequately documented (Table 1). In some cases, the identification was made through histological examination or direct visualization of the mites, whereas others relied solely on clinical observations without any morphological confirmation.

Despite its apparently cosmopolitan distribution, the life cycle of *L. cysticola* is still poorly understood. Neither its complete developmental stages nor its routes of transmission have been definitively clarified. The aim of the present study is to propose a plausible reconstruction of the life cycle of the mite by comparing observations made in two naturally infested backyard hens with previously published data. The different developmental stages of the mite (larvae, nymphs, and adults) were characterized through both morphological and histopathological analysis, in order to better understand its migratory behavior and adaptive strategies within the avian host.

## 2. Materials and Methods

Two adult laying hens underwent complete necropsy [26,27] at the Department of Veterinary Medicine at the University of Perugia after being culled by the owner due to the appearance of crusts on the legs. The birds originated from a rural flock located near Assisi (Umbria, Central Italy), comprising approximately 30 chickens. The poultry house was situated on the slopes of Monte Subasio, in an area with predominantly clayey soil and subject to frequent rainfall, which resulted in the formation of stagnant water and high ambient humidity. The shelter structures were made of non-elevated wood and prone to water infiltration, particularly during adverse weather conditions. During the winter, internal temperatures ranged between −1 °C and +5 °C, with relative humidity levels between 90% and 95%, creating conditions that encouraged gregarious behavior among the animals. At necropsy, both hens exhibited multifocal subcutaneous nodular lesions, particularly evident in the pectoral region. The nodules, well-demarcated and yellowish-white in color, were sampled and fixed in 10% neutral buffered formalin for histological examination. The samples were processed routinely, embedded in paraffin wax, sectioned at 3 μm, and stained with Hematoxylin and Eosin. Microscopic examinations were carried out using an Olympus BX50 microscope (Olympus Corporation, Tokyo, Japan).

For parasitological analysis, crust scrapings and subcutaneous tissue samples were collected. All nodular samples were stored at −20 °C and subjected to a systematic microscopic investigation lasting over one year. This analysis aimed to determine the anatomical distribution of *L. cysticola* at various developmental stages and to assess the localization of micronodules in different body regions. Each sample was labelled with its anatomical origin, and these data were correlated with the developmental stage of the mites to hypothesize the parasite’s life cycle.

Mites isolated from crusts, cysts, and tissue samples were cleared using lactic acid and mounted on slides in heated Berlese solution. Observations were conducted under a light microscope (Olympus CX31, Olympus Corporation, Tokyo, Japan) at 4×, 10×, and 40× magnifications [28]. Morphometric measurements were performed on five randomly selected adult females. Mite identification and classification followed the morphological criteria described by Fain [8].

## 3. Results

Upon external inspection, both hens displayed typical crusty lesions on the legs, consistent with scaly leg mite infestation. The parasitological examination of the crusts revealed a high number of mites identified as *Knemidokoptes mutans* (Acarina: Knemidokoptidae), the causative agent of knemidocoptic mange in birds. This condition is commonly observed in older birds, especially those reared in rural or backyard environments.

**Figure 1:**
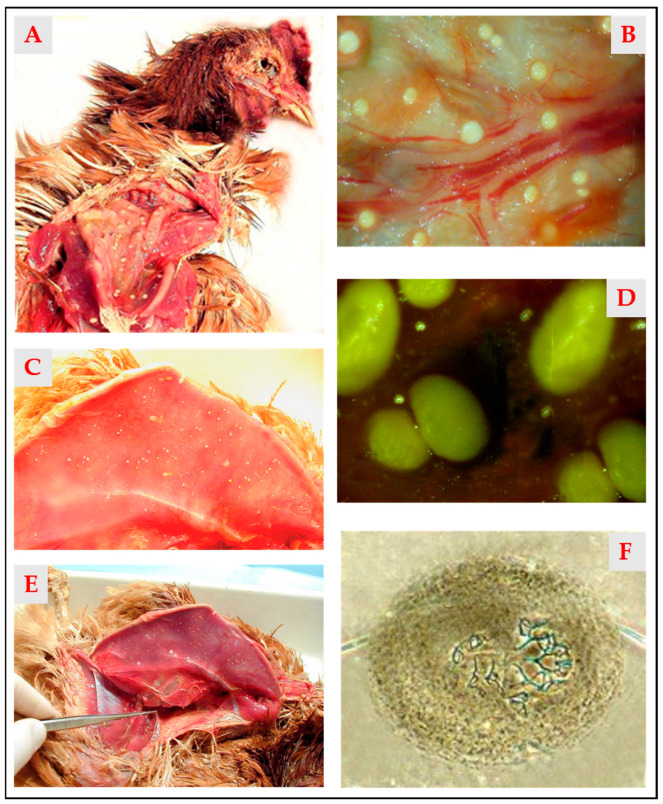
Typical nodular appearance of avian scaly leg mite infestation in chickens. (**A**)—Nodules in the peritracheal area; (**B**)—Subcutaneous nodules; (**C**)—Nodules in the pectoral area; (**D**)—Visceral nodules of various sizes; (**E**)—Characteristic subcutaneous material with a consistency similar to egg whites; (**F**)—A nodule containing a degenerate adult of *Laminosioptes cysticola* inside—40×. Photos (**A**–**E**) were taken with a OM SYSTEM TG-7 camera (OM Digital Solutions Corporation, Tokyo, Japan).

The necropsy revealed numerous white nodules, ranging from 0.5 to 4 mm in diameter, located in the subcutaneous tissue and various visceral serosal surfaces (Figure 1A–E).

In particular, a gelatinous, transparent, egg-white-like hyaline substance was observed in the thoracic and femoral subcutis, as previously described by Principato et al. [9] and Smith et al. [10], associated with a high concentration of free adult mites. The parasitological analysis of the nodules—especially those in early stages, not yet affected by calcification—revealed the presence of viable mites (Figure 1F).

The systematic examination of the tissues enabled the isolation of multiple developmental stages of the mite, both in the subcutaneous tissue and the serosal surfaces of internal organs. The mites were identified as *Laminosioptes cysticola*, based on the morphological criteria described by Fain [2].

**Figure 2:**
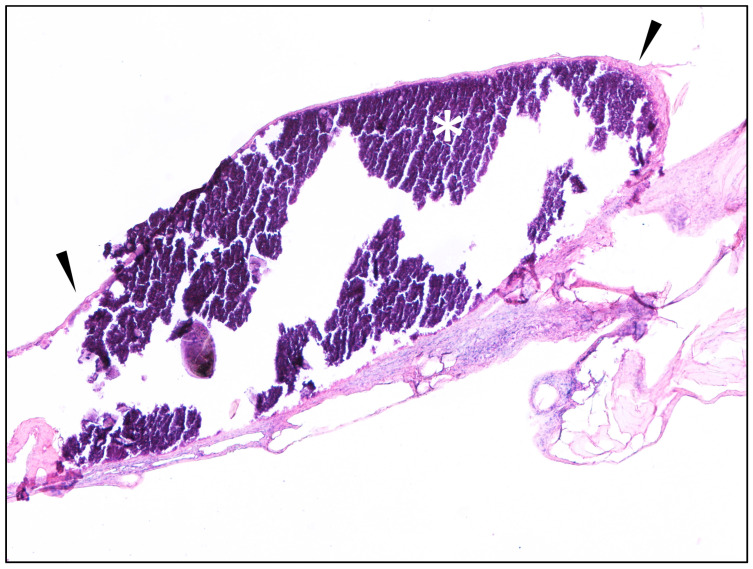
Histological findings of the examined lesions. A subcutaneous oval nodule composed of central mineralization phenomena (asterisk *) and peripheral fibrous capsule (black arrowhead) is seen immersed in fibrous connective tissue and normal adipose tissue lobules (Hematoxylin and Eosin, 40×).

Histological sections showed mature connective tissue with normal lobules of adipose tissue. Among collagen fibers, a nodule measuring approximately 2 × 1 mm was observed, characterized by basophilic crystalline material (indicative of mineralization) surrounded by fibroblasts embedded in an eosinophilic collagen matrix forming a fibrous capsule (Figure 2). Scattered peripheral macrophages were also noted. No viable parasites were observed in these sections. However, the lesion morphology was consistent with chronic infestations.

### Morphological Observations and Biological Considerations

The morphometric data of five adult females are presented in Table 2.

**Figure 3:**
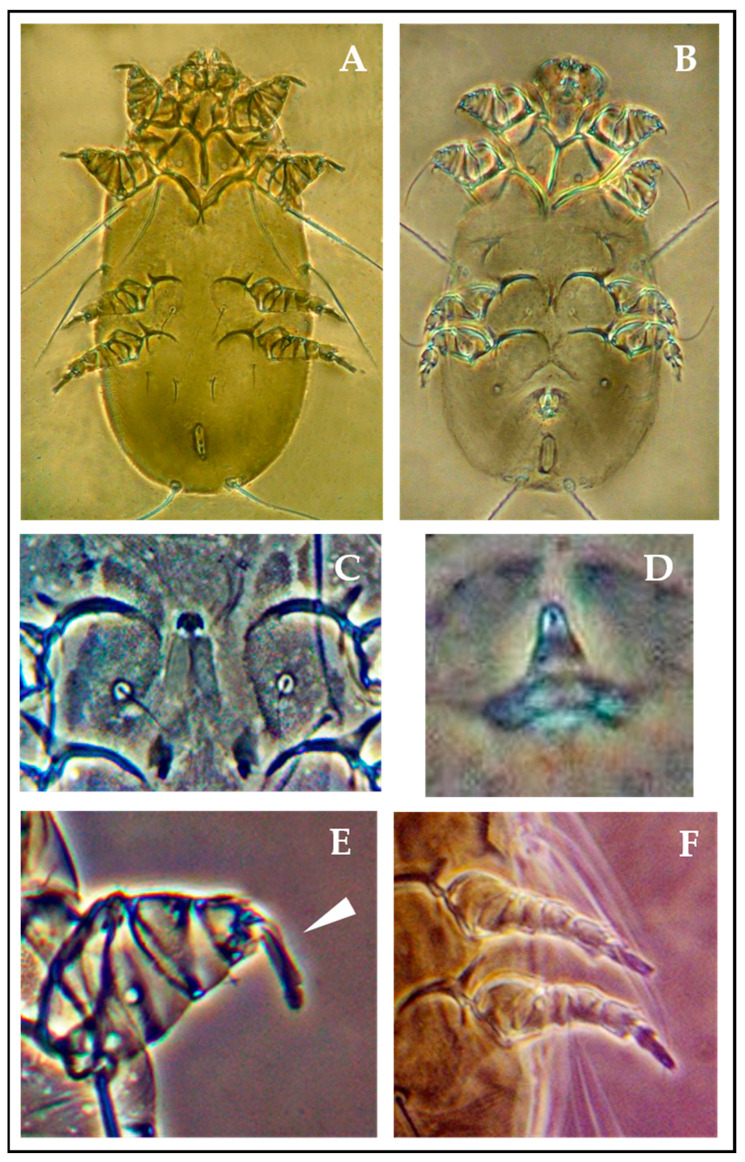
Anatomical features of *Laminosioptes cysticola*. (**A**)—Adult female—10×; (**B**)—Adult male—10×; (**C**)—Female genitalia—40×; (**D**)—Male aedeagus—40×; (**E**)—Leg II with long pretarsus (white arrowhead)—40×; (**F**)—Leg III and IV—40×.

Adult *L. cysticola* mites (Figure 3A,B) exhibit an elongated idiosoma (total body length is 280 μm), widest in the podosomal region (121 μm) and tapering anteriorly (99 μm) and posteriorly (88 μm). The propodosoma is sclerotized and reinforced by robust, closely aligned anterior epimeres, forming an internal support structure. The dorsal cuticle is finely granular and bears two pairs of stout lateral setae (l1 and l2), measuring 116 μm and 99 μm, respectively, followed posteriorly by two long caudal setae (d4) measuring 154 μm.

The gnathosoma, trapezoidal and broader than tall (44 × 33 μm), displays subtriangular lateral projections at the base of the articulated palps, which are equipped with small hooks. Ventrally, the stylet-like chelicerae are located within the gnathosomal cavity.

The first two pairs of legs are short, stout, and adapted for tissue penetration, terminating in long pretarsi of 13.2 μm (Figure 3E). In contrast, the third and fourth legs are longer and more slender (Figure 3F), located ventrolaterally on the hysterosoma, and bear posterior epimeres that are separated from each other and from the anterior epimeres.

These morphological traits—elongated idiosoma, sclerotized propodosoma, robust anterior legs, and armored gnathosoma—suggest adaptive evolution for effective migration through subcutaneous tissue and infiltration into the thoraco-abdominal cavity and visceral organs. These adaptations are comparable to those found in other tissue-dwelling mites, such as *Sarcoptes*, *Knemidocoptes*, *Hypodectes*, and *Demodex*.

Marked sexual dimorphism was evident: males possess a curved aedeagus protruding ventrally from the genital opening, while females exhibit a crescent-shaped epigynum (Figure 3C,D). The parasitological analysis revealed a clear majority of adult females; however, no eggs or ovigerous females were observed, supporting the hypothesis of larviparity. Larvae and first- and second-stage nymphs were found predominantly in the periesophageal region, while a few immature stages were isolated from the subcutis of the limbs. Adult mites, accounting for most of the specimens, were mainly found in the subcutaneous tissue and on the surfaces of internal organs such as the intestines, kidneys, air sacs, heart, and liver.

## 4. Discussion

The microscopic analysis revealed two distinct populations of adult mites based on anatomical localization. Specimens retrieved from the subcutaneous tissue appeared fully mature, strongly sclerotized, and sexually differentiated, whereas those found on the serosal surfaces of internal organs were less sclerotized, suggesting they were either recently molted adults or still in late developmental stages. This distribution supports the hypothesis of a structured migratory pattern. The findings support the thesis that the parasite’s life cycle begins in the cervical region, where larvae and nymphs develop within the loose connective tissues surrounding the trachea and esophagus. Once fully developed, adult mites migrate into the thoracic and abdominal cavities, colonizing the serosal surfaces of visceral organs. The return to the pectoral subcutis likely occurs only in reproductively competent individuals. The high degree of sclerotization in subcutaneously located adults, along with the exclusive presence of sexually differentiated females in this region, suggests that mating predominantly occurs in the pectoral subcutis. Fertilized females, being larviparous, would release larvae in the cervical area, where the skin is thinner and less resistant. This would facilitate both tissue penetration and potential transmission to other birds through prolonged close contact.

Infestation by *L. cysticola*, as described in this study, is typically observed in rural backyard poultry farms, characterized by suboptimal hygiene, the presence of synanthropic birds (e.g., pigeons), and extended lifespans of reproductive birds. Such conditions may allow for the slow development of chronic infestations that are clinically silent or difficult to detect.

In accordance with previous reports [9,10], histological findings revealed chronic granulomatous reactions surrounding the mites, often in the absence of viable organisms, supporting the interpretation of these lesions as terminal. Due to its chronic progression, this parasitosis may remain clinically inapparent until advanced stages or when associated with other debilitating conditions.

From an epidemiological standpoint, interindividual transmission appears to be an uncommon event. Three major limiting factors can be identified: (1) the rarity of males, comprising the smallest number of the total population, which limits mating opportunities; (2) the requirement for close physical contact to enable the transfer of fertilized females to a new host; and (3) the necessity for environmental conditions favorable to survival and contact, such as relative humidity above 90% and temperatures below 5 °C, which induce gregarious behavior in birds. In the present study, co-infestation with knemidokoptic mange—an intensely pruritic and debilitating condition—may have contributed to mite proliferation and dissemination by promoting stress and immunosuppression.

### Hypothetical Life Cycle of Laminosioptes Cysticola

**Figure 4:**
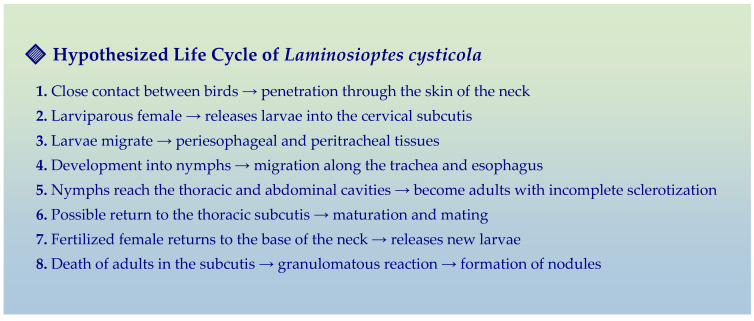
Putative life cycle of *Laminosioptes cysticola*.

Based on both our observations and literature data [2,8,9], the following hypothetical life cycle is proposed (Figure 4):

✓ Point of entry: The skin of the neck, particularly thin and poorly protected by underlying musculature [5], is likely the main access site.
✓ Reproduction: Mating occurs in the pectoral subcutis, where larviparous females give birth to mobile larvae.
✓ Initial migration: Larvae migrate into the loose connective tissues surrounding the trachea and esophagus, transforming into nymphs within 24–48 h.
✓ Colonization: Nymphs migrate along the esophagus and trachea to reach the thoracic and abdominal cavities, where they complete development into adults.
✓ Dissemination: Adults may either return to the subcutis to reproduce or reach the skin surface for transmission to another host.

Environmental elimination is potentially supported by the high sclerotization of mature adults, which could allow short-term survival in moist environments such as bedding or nests. This hypothesis is further supported by the existence of *Laminosioptes hymenopterus*, a closely related species that colonizes the skin and feathers [29].

In the terminal phase, adult mites die within the subcutis, where they induce a granulomatous inflammatory response resulting in the formation of calcified nodules. During our investigation, many micronodules contained no visible parasites but instead enclosed fibrotic aggregations around a central mite, suggesting that such structures may develop independently in response to mite-derived proteinaceous exudates, rather than exclusively as post-mortem encapsulations.

Finally, the confirmation of larviparity—indicated by the complete absence of eggs and the identification of prelarval stages—constitutes a critical biological feature of *L. cysticola*, one that remains poorly understood or misreported in several sources.

Due to its sporadic occurrence and a still poorly understood life cycle, diagnosing *L. cysticola* is not always straightforward, and the correct identification of this parasite remains challenging. Numerous reports in the literature have misidentified this mite, often confusing it with *Hypodectes propus*—another mite responsible for subcutaneous acariasis, especially in pigeons—or with larval forms of knemidokoptic mites, likely due to the limited availability of detailed morphological descriptions. The diagnostic keys provided by Fain [2,8] remain among the most comprehensive, yet they are seldom cited in general avian pathology texts.

## 5. Conclusions

Nodular acariasis caused by *L. cysticola* is a rare parasitic condition that nevertheless warrants attention due to its pathological, ecological, and diagnostic implications. The data obtained suggest that environmental, immunological, and behavioral factors contribute significantly to the pathogenesis and clinical expression of the infestation.

In particular, environmental conditions such as low temperatures and relative humidity above 90% favor gregarious behavior in birds, increasing the likelihood of direct transmission. In rural settings, where poultry housing is often wooden, non-elevated, and prone to moisture accumulation, such conditions are far from uncommon.

The parasite appears to develop within a gelatinous, albumin-like matrix that maintains adequate hydration for survival within the host. It is plausible that *L. cysticola* can survive outside the host only in conditions of very high humidity, which may, under specific circumstances, permit successful transmission and completion of the life cycle on a new host.

Advanced age and potential immunosuppression—possibly exacerbated by concurrent conditions such as knemidokoptic mange, as observed in this study—are crucial predisposing factors for heavy infestations. These conditions may facilitate deeper tissue migration and contribute to the silent progression of the disease.

Although nodular acariasis does not pose a significant economic threat in small-scale or rural poultry farming—especially since affected older animals are rarely destined for human consumption—the potential health implications of consuming such birds remain to be fully clarified.

In light of these considerations, *L. cysticola* deserves further study to elucidate its life cycle, transmission pathways, and potential zoonotic relevance. The development of more sensitive diagnostic tools and the wider dissemination of accurate morphological identification keys are essential for improving the recognition and management of this insidious and neglected acariasis.

## Figures and Tables

**Table 1:** Records of *Laminosioptes cysticola* across different geographical areas of the world and the identification method used.

Reference	Country	Identification Method			
		Not Specified	Presence of Nodules	Mite Isolation and Microscopic Identification	Histology
Roveda and Ringuelet, 1947 [1]	Argentina	x			
Fain, 1956 [2]	Rwanda		x	x	
Cassidy and Ketter, 1965 [3]	USA		x	x	x
Cvetkovic et al., 1965 [4]	Serbia	x			
Kaliner, 1970 [5]	Kenya		x		x
Morrow, 1986 [6]	Australia	x			
Amure and Stuart, 1977 [7]	UK		x		
Fain, 1981 [8]	Belgium, Rwanda		x	x	
Principato et al., 1991 [9]	Italy		x	x	x
Smith et al., 1997 [10]	USA		x	x	x
Toro et al., 1999 [11]	Chile		x		
Smolska-Szymczewska and Paszowska, 2000 [12]	Poland		x		x
Reina et al., 2002 [13]	Spain		x		
Chen and Fan, 2003 [14]	China		x		
Mukaratirwa and Hove, 2009 [15]	Zimbabwe	x			
Eslami et al., 2009 [16]	Iran		x		
Soriano-Vargas et al., 2010 [17]	Mexico		x		x
Martins et al., 2010 [18]	Brazil		x	x	x
Herpich et al., 2012 [19]	Brazil		x		x
Radfar et al., 2012 [20]	Iran		x		
Lawal et al., 2016 [21]	Nigeria	x			
Taracena Rivera, 2019 [22]	Guatemala		x	x	
Kaboudi et al., 2019 [23]	Tunisia		x		
Heath et al., 2022 [24]	New Zealand	x			
Grist et al., 2022 [25] *	UK		x		x
Present study	Italy		x	x	x

* Only study in which molecular identification by PCR was attempted, with negative results.

**Table 2:** Morphometric data of *Laminosioptes cysticola* female (µm).

**Gnathosoma** (µm)	Width	Height	Chelicerae	Palps
	44	33	22	24
**Leg Number** Length (µm)	I	II	III	IV
	48	48	55	55
**Epimere Number** Length (µm)	I	II	III	IV
	41.8	41.8	26.4	26.4
**Idiosoma** (µm)	Length	Opisthosoma Width	Podosoma Width	Propodosoma Width
	247	88	121	99
**Setae** Length (µm)	Paranal (pa)	Opisthosomal (d4)	Lateral (l1)	Lateral (l2)
	24.2	154	116	99

## Data Availability

The original contributions presented in this study are included in the article. Further inquiries can be directed to the corresponding author.
